# Perinatal Western-style diet alters serotonergic neurons in the macaque raphe nuclei

**DOI:** 10.3389/fnins.2022.1067479

**Published:** 2023-01-10

**Authors:** Geoffrey A. Dunn, Jacqueline R. Thompson, A J Mitchell, Samantha Papadakis, Matthew Selby, Damien Fair, Hanna C. Gustafsson, Elinor L. Sullivan

**Affiliations:** ^1^Department of Human Physiology, University of Oregon, Eugene, OR, United States; ^2^Department of Psychiatry, SUNY Upstate Medical University, Syracuse, NY, United States; ^3^Division of Neuroscience, Oregon National Primate Research Center, Beaverton, OR, United States; ^4^Department of Psychiatry, Oregon Health & Science University, Portland, OR, United States; ^5^Masonic Institute of Child Development, University of Minnesota School of Medicine, Minneapolis, MN, United States

**Keywords:** maternal obesity, nutrition, Western-style diet, serotonin, VGLUT3, dorsal raphe, non-human primates

## Abstract

**Introduction:**

The neurotransmitter serotonin is a key regulator of neurotransmission, mood, and behavior and is essential in neurodevelopment. Dysfunction in this important neurotransmitter system is connected to behavioral disorders such as depression and anxiety. We have previously shown that the developing serotonin system is sensitive to perinatal exposure to Western-style diet (WSD).

**Methods:**

To advance our hypothesis that perinatal WSD has a long-term impact on the serotonergic system, we designed a fluorescent immunohistochemistry experiment using antibodies against tryptophan hydroxylase 2 (TPH2) and vesicular glutamate transporter 3 (VGLUT3) to probe protein expression in the raphe subnuclei in 13-month-old Japanese macaques (*Macaca fuscata*; *n* = 22). VGLUT3 has been shown to be coexpressed in TPH2+ cells in the dorsal raphe (DR) and median raphe nucleus (MnR) of rodent raphe nuclei and may provide information about the projection site of serotonergic fibers into the forebrain. We also sought to improve scientific understanding of the heterogeneity of the serotonin production center for the central nervous system, the midbrain raphe nuclei.

**Results:**

In this immunohistochemical study, we provide the most detailed characterization of the developing primate raphe to date. We utilize multi-level modeling (MLM) to simultaneously probe the contribution of WSD, offspring sex, and raphe anatomical location, to raphe neuronal measurements. Our molecular and morphological characterization revealed that the 13-month-old macaque DR is remarkably similar to that of adult macaques and humans. We demonstrate that vesicular glutamate transporter 3 (VGLUT3), which rodent studies have recently shown can distinguish raphe populations with distinct projection targets and behavioral functions, likewise contributes to the heterogeneity of the primate raphe.

**Discussion:**

This study provides evidence that perinatal WSD has a long-term impact on the density of serotonin-producing neurons, potentially limiting serotonin availability throughout the brain. Due to the critical involvement of serotonin in development and behavior, these findings provide important insight into the mechanisms by which maternal nutrition and metabolic state influence offspring behavioral outcomes. Finally, these findings could inform future research focused on designing therapeutic interventions to optimize neural development and decrease a child’s risk of developing a mental health disorder.

## 1. Introduction

Serotonin is critical for the healthy development of the central nervous system where it actively shapes neuronal networks and coordinates behavioral response and cognition (Kraus et al., 2017; Bacqué-Cazenave et al., 2020). Dysfunction in the central serotonergic system contributes to behavioral pathologies such as anxiety, major depressive disorder, and obsessive-compulsive disorder (Ciranna, 2006; Kraus et al., 2017; Sinopoli et al., 2017). Considering its importance, a surprisingly small number of neurons are responsible for producing the serotonin transmitted throughout the brain. These neurons reside in the raphe nuclei of the midbrain and contain the enzyme tryptophan hydroxylase 2 (TPH2) which is responsible for synthesizing endogenous serotonin used to signal between neurons.

Previous work in our lab has shown that disruption of TPH2 synthesis in the raphe corresponds to decreased serotonin delivery in the cortex and behavioral dysregulation in developing non-human primates (Thompson et al., 2017; Thompson et al., 2018). These outcomes resulted from developmental exposure to a Western-style diet (WSD) and the associated metabolic responses, part of a larger body of work demonstrating that maternal diet and metabolic state have long-lasting influences on offspring behavior and neurodevelopment (Rivera et al., 2015; DeCapo et al., 2019). Recent work in rodents has emphasized the susceptibility of the serotonin system to dietary influences during development (Gawlińska et al., 2021). As such, identifying how and why serotonergic signaling is impaired during developmental insult is highly relevant to researchers and for public health.

The TPH2 populations that primarily supply the forebrain with serotonin are the dorsal raphe (DR), largely confined to the periaqueductal gray (PAG), and the more ventrally located median raphe. Modern research has highlighted the diversity of the raphe nuclei, relying heavily on rodent models (Okaty et al., 2019). The DR is organized into smaller subnuclei arranged along the rostral-caudal axis of the hindbrain (Alonso et al., 2013). These subnuclei are delineated by anatomical boundaries, developmental lineage, co-receptor expression, morphological characteristics, and electrophysiological properties (Calizo et al., 2011).

Recent advances in single cell characterization and projection tracing have revealed that the heterogenous phenotypes of these subnuclei correspond to populations of cells with distinct projection targets (Okaty et al., 2015; Fernandez et al., 2016; Ren et al., 2018; Ren et al., 2019). One particular molecular marker, VGLUT3, has emerged as a particularly straightforward and informative indication of DR subnuclei. In humans and rodents, populations of TPH2 positive neurons in the raphe co-express VGLUT3 to varying degrees (Hioki et al., 2010; Vigneault et al., 2015; Deneris and Gaspar, 2018).

This heterogeneity is associated with behavioral regulation. VGLUT3+ DR projections concentrate in behaviorally relevant brain regions like the medial prefrontal cortex and amygdala (Amilhon et al., 2010; Prouty et al., 2017; Ren et al., 2018; Sengupta and Holmes, 2019). Loss of VGLUT3 impacts behavioral response, with one study showing VGLUT3 null mice exhibited increased anxiety-like behaviors during development and adulthood (Amilhon et al., 2010). Unfortunately, apart from one study establishing the presence of VGLUT3 in the human DR, all of the evidence implicating VGLUT3 with the behaviorally relevant functions of the DR come from rodent models. To date there have been no studies examining the expression of VGLUT3 in the non-human primate DR.

In the current study, we labeled midbrain tissue sections with anti-TPH2 and anti-VGLUT3 antibodies to probe the diversity of the juvenile Japanese macaque (*Macaca fuscata*) raphe nuclei. Examining cells across the rostral-caudal extent of the raphe, we looked across anatomical boundaries at morphological characteristics such as cell area and shape, cell density, VGLUT3 identity, and TPH2 intensity to examine how serotonergic neuron diversity in the macaque relates to that reported in rodent literature. We hope that this study can be a resource for future studies of serotonergic development in primates. We simultaneously leveraged the WSD model established in our research group to investigate how perinatal WSD impacts serotonergic integrity across raphe subnuclei. We examined three diet groups: animals exposed to a single diet from gestation through post-weaning, either the control diet or WSD, and animals that were exposed to WSD during gestation and lactation but received a control diet intervention from post-weaning onward. We hypothesized that perinatal WSD would decrease TPH2 availability across the raphe. Additionally, because of our groups previous *tph2* mRNA findings (Thompson et al., 2017), we hypothesized that TPH2 protein in animals that were switched to a control (CTR) diet at weaning would remain reduced. We predicted that compensatory changes in VGLUT3 expression may coincide with TPH2 disturbances.

## 2. Materials and methods

### 2.1. Animals

All animal procedures were in accordance with National Institutes of Health guidelines on the ethical use of animals and were approved by the Oregon National Primate Research Center (ONPRC) Institutional Animal Care and Use Committee.

Japanese macaques (*M. fuscata*) used in this study were born naturally into social groups and kept with their mothers until weaning at 8.43 ± 0.90 months of age. After weaning, animals were housed in mixed-sex social groups with 4–7 other age-matched Japanese macaques and 1–2 unrelated adult females.

### 2.2. Diet characterization

Two diets were administered to animals in this study: the WSD and a CTR diet. The WSD (TAD Primate Diet no. 5LOP, Test Diet, Purina Mills, St. Louis, MO, United States) provides approximately 36.6% of calories from fat, which is in line with the fat and saturated fat content of the typical Western-style, American diet. Alternatively, the CTR diet (Monkey Diet no. 5000; Purina Mills) provides approximately 14.7% of calories from fat. The carbohydrate sources differed between the two diets, with sugars (primarily sucrose and fructose) comprising 18.94% of the WSD but only 3.14% of the CTR diet. All animals fed the WSD were also given calorically dense treats once per day (Thompson et al., 2017).

Animals were assigned to a diet group based on their source of nutrition in two life-stages: maternal (pre-weaning) diet and post-weaning diet. In the case of maternal WSD, pregnant adult females had consumed the WSD diet for at least 1 year before producing offspring considered in this study. In the case of the maternal CTR diet, adult females had been fed CTR chow for their entire lives. The offspring were exposed to the maternal diet *in utero*. Following birth, all animals maintained their same diet such that the offspring received continued nutrition from the maternal diet in the form of breastmilk. Most offspring began independently ingesting the maternal diet by 4 months of age and by 6 months of age this was their primary food source.

When animals were weaned, most juveniles continued their maternal diet post-weaning (CTR/CTR or WSD/WSD). Some maternal WSD animals received a diet intervention and switched to the CTR diet post-weaning (WSD/CTR). In this way we can examine the effect of nutrition during early development (maternal diet) or later development (post-weaning diet). A total of 24 animals were used in this study: 8 C/C (4 female), 8 W/C (4 female), 8 W/W (4 female).

### 2.3. Tissue collection

Brain tissue was obtained from the Obese Resource tissue bank (P51 OD011092). Tissue utilized in this experiment were part of a larger more comprehensive study that examined many different tissue systems there may be impacted by maternal diet. At 13.25 ± 0.71 months of age the juveniles were euthanized and brain tissue was collected. Euthanasia was performed by ONPRC Necropsy staff and adhered to American Veterinary Medical Association Guidelines on Euthanasia in Animals and ONPRC standard operating procedures and guidelines. Animals were sedated with ketamine HCl (15–25 mg/kg i.m.) for transport to necropsy suite and then deeply anesthetized with a surgical dose of sodium pentobarbital (25–35 mg/kg i.v.). Once sufficient anesthetic depth was reached, animals were exsanguinated from the aorta while cerebral perfusion was performed *via* the carotid artery. Perfusion consisted of an initial flush of 0.5–1 liter of 0.9% heparinized saline followed by 4% paraformaldehyde (PFA, approximately 1–2 L) buffered with sodium phosphate (NaPO4, pH 7.4) until tissue was fixed. The brain was then removed, regionally partitioned, and placed in 4% PFA for 24 hours at 4°C to complete fixation. Brain tissue blocks were then transferred to 10% glycerol buffered with NaPO4 for 24 hours and finally transferred to 20% glycerol solution for 72 hours. Tissue blocks were frozen in −50°C 2-methylbutane and then stored at −80°C until sectioning. Coronal sections (25 μm) of the midbrain were collected in 1:24 series using a freezing microtome and stored in cryoprotectant at −20°C until immunohistochemistry was performed.

### 2.4. Tissue selection

Detailed stereological assessment of the entire DR was not possible to due to the use of some tissue sections from these animals in previous experiments. While our sections could not be used to quantitatively reconstruct the entire DR, the sections used in this experiment for all animals spanned the rostro-caudal progression of the raphe.

**FIGURE 1:**
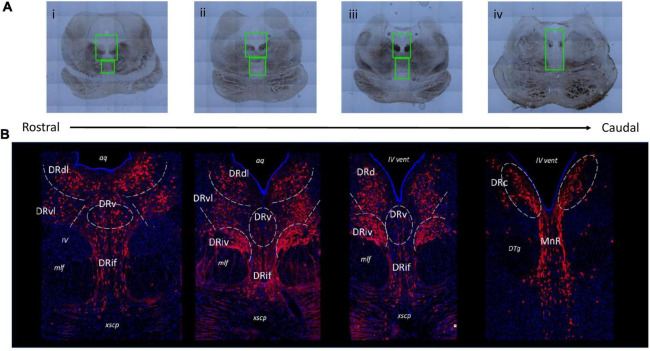
Representative images of various subnuclei distinctions across the rostral-caudal axis of the greater raphe nuclei. Distinctions were made based on gross anatomical makers such as the size and shape of the periaqueductal gray (PAG) and cerebral aqueduct (aq)/fourth ventricle (iv vent), the presence and location of medial longitudinal fasciculus (mlf), and the presence of fibrous pontine tissue. **(A)** Example overview images indicating slide scanner imaging areas. Green boxes indicate locations of 20× magnification images that were used in analysis. **(B)** Representative images of juvenile Japanese macaque midbrain sections indicating the subnuclei divisions used in subsequent analysis. *Gray* dashed lines indicate nuclei boundaries the experimenter identified and where rectangular bounding box was placed to produce separate images for each subnucleus. The lateral nuclei (DRdl and DRvl) and the DRiv were separated into left and right components, but only left components are labeled for visualization purposes. Blue: 4′,6-diamidino-2-phenylindole (DAPI), red: tryptophan hydroxylase 2 (TPH2). DRd, dorsal nucleus of the dorsal raphe; DRdl, dorsolateral nucleus of the dorsal raphe; DRv, ventral nucleus of the dorsal raphe; DRvl, ventrolateral nucleus of the dorsal raphe; DRiv, fourth nerve nucleus of the dorsal raphe; DRif, interfascicular nucleus of the dorsal raphe subnucleus; MnR, median raphe nucleus; xscp, cerebellar decussation; DTg, dorsal tegmental nucleus.

Brain sections could be identified as corresponding to rostral, medial, and caudal raphe based on gross anatomical markers. The most informative anatomical markers were the size and shape of the PAG and cerebral aqueduct (*aq*)/fourth ventricle (*iv vent*), the presence and location of medial longitudinal fasciculus (*mlf*), and the presence of fibrous pontine tissue. This selection technique was refined in pilot experiments using a set of six additional juvenile brains that were not included in the experimental dataset. This pilot group consisted of 3 C/C (2 females), 1 W/C (0 females), and 2 W/W (1 female) whose brain sections represented the range of sectioning angles and section sizes present in the experimental group. Through these pilot studies we determined that classification of the floating tissue section along a custom 10-point rostral-caudal scale based on anatomical markers matched well with the actual classification of the section based on immunofluorescent staining of TPH2 populations. The full rostro-caudal extent of the raphe could be represented with 12–14 sections collected from the midbrain. The final number of sections included per animal in the experimental dataset was as follows: C/C = 8–14 sections per animal (median: 12), W/C = 9–14 sections per animal (median: 11), W/W = 10–14 sections per animal (median: 13). Example images illustrating the section heterogeneity across the raphe are presented in Figure 1.

### 2.5. Immunohistochemistry

Tissue sections were removed from cryoprotectant and transferred to phosphate buffer solution (PBS) in netter wells in a six-well plate. Unless otherwise stated, all steps occurred at room temperature. Sections underwent four 5-min washes, using fresh PBS for each wash. Tissue was then blocked for 1 hour in 5% normal donkey serum (NDS; Millipore-Sigma Cat# S30-100ML), 0.4% Triton-X 100 (Fisher Bioreagents Cat# BP151-500) in PBS. Sections were then transferred to primary antibody solution (2% NDS in PBS) containing the following primary antibodies: 1:500 anti-TPH2 goat (Abcam Cat# 121103), 1:100 anti-VGLUT3 mouse (Abcam Cat# 134310). Sections were incubated at room temperature at 40 RPM for 1 hour and then transferred to 4°C where they were kept for 22 hours.

After primary incubation, sections were washed three times (5-min each at 40 RPM in fresh PBS). Tissue was then transferred to secondary antibody solution (2% NDS in PBS) containing: 1:300 donkey anti-goat TRITC (Abcam Cat# 6882), 1:500 donkey anti-mouse Alexa 647 (Abcam Cat# 150107). Secondary incubation occurred at 40 RPM for 1 hour, followed by three more washes (5-min each at 40 RPM in fresh PBS). Nuclei were then labeled with DAPI (4′,6-diamidino-2-phenylindole; 3 μM in PBS; ThermoFisher Cat# D3571) for 3 min at 40 RPM before two final 5-min washes in fresh PBS. Sections were then moved into fresh PBS before being mounted onto gelatin-subbed slides. Slides were finally coverslipped with 100 μL Prolong Gold (ThermoFisher Cat# P36930) and 1 mm coverslips (Thermo Scientific Cat# 6776215). Slides were left to cure overnight before they were sealed with nail polish and stored in 4°C until imaging.

### 2.6. Imaging

Images were taken using the Olympus VS110 slide-scanning microscope. Images were collected at 20×. Channel settings were as follows: DAPI exposure = 100 ms, intensity = 100%, dynamic range: 300–8,000; FITC exposure = 250 ms, intensity = 100%, dynamic range: 750–6,000; TRITC exposure = 200 ms, intensity = 100%, dynamic range: 1,000–3,000; Cy5 exposure = 500 ms, intensity = 90%, dynamic range: 500–1,500.

The pilot studies referenced above also determined the imaging area bounding box, based on anatomical landmarks present in brightfield images. For rostral and medial sections, or any section clearly containing the cerebellar decussation (*xcsp*), a single DR image was collected (Figure 1Ai–iii). The upper limit of the DR image area was slightly above the widest point of the PAG and always included a small portion of the aq. The image area extended ventrally to the upper quarter or middle of the *xcsp*, which marked the lower limit. The width of the DR imaging area was defined by the far edges of the *mlf*. For caudal sections (Figure 1A iv), a single image was taken that contained both the caudal DR (DRc) and the median raphe. The upper limit began at approximately half the height of the PAG and the lower limit was the upper boundary of the pons. The width was determined by the outer edge of the densest dorsal tegmental nuclei bundles (*DTg*). Focal spots were determined after image area selection, with approximately 6 evenly spaced focal spots selected in the PAG of rostral and medial sections, and only 2 in the PAG for caudal sections. Between 2–3 focal spots were placed in the interfascicular region, and the IV nerve bundles received 1 focal each. Once all focal spots were determined, imaging commenced with the Olympus system prompting the experimenter to manually focus (using the DAPI channel) at each of the determined focal spots. The entire image area was then collected by the scanning microscope (Figure 1B).

### 2.7. Subnuclei division

Given the lack of serial sections, plus the variation in tissue size and sectioning angle, we opted to not perform true stereological quantification on the entire DR. Rather, we adopted a user-defined sampling method to isolate image regions for quantification. The criteria used to define these quantification regions are derived from literature descriptions of the diverse subnuclei within the DR.

We found that the juvenile macaque raphe mapped consistently to the adult human DR (Baker et al., 1990; Figure 1B). Based on the histological location and cell morphology outlined in Baker et al., 1990, we identified the dorsal DR (DRd), ventral DR (DRv), ventrolateral DR (DRvl), interfascicular DR (DRif), DRc, a IV nerve, mlf subnucleus (DRiv), and MnR (Bethea and Reddy, 2012). We further segregated the rostral DRd as its own subnucleus, since we consistently saw (1) rostral DRd positions were more lateral and (2) rostral DRd exhibited distinct cells size and density patterns that medial DRd and DRc. Owing to its more wing-like population, we called this the dorsolateral DR (DRdl).

Within the CellSens program we used rectangular bounding boxes to isolate the TPH2 neurons that appeared in these stereotyped locations. We generated distinct images for each of the eight subnuclei: DRdl, DRvl, DRv, DRd, DRc, DRif, DRiv, and MnR. The DRdl, DRvl, DRd, DRc, and DRiv all were present in bilaterally symmetric clusters, so two cropped images of each of these subnuclei were generated from each initial DR image.

### 2.8. Image processing

FIJI (Schindelin et al., 2012) for ImageJ (Rueden et al., 2017) software was used to develop an in-house macro to combine automated and manual TPH2 cell measurement. The macro steps are summarized here and are available from the authors upon request.

First, the macro performed a minimum filter (5 pixels) of the raw TPH2 image and then averaged the minimum filtered image with the original raw image. For all images except for DRc, the Triangle thresholding algorithm was then applied to the averaged image. Due to the proximity of the DRc to the tissue border of the iv vent and the resulting increase in background for those images, the MaxEntropy thresholding algorithm was used for DRc images only. The subsequent processing steps were consistent for all images.

After thresholding, raw grayscale image was overlaid atop the resultant binary image such that the detail of the grayscale image was preserved but the thresholded portions of the image appeared faintly red. An experimenter (blind to all experimental conditions and tissue source) used these thresholding guides to manually count the TPH2 cells in the image. Immediately after counting, experimenters were prompted to review the thresholded cell selection. The paint tool was used to ensure that each cell was properly segmented and considered separate from its neighbor. The final binary image was saved and imported into CellProfiler for quantification.

FIJI ImageJ software was also used to determine the threshold of VGLUT3 signal. Display minimum and maximum were set to fixed values for all raw images and the 16 color LUT was applied to visually identify the intensity range that reliably measured true positive signal with little background. This method indicated that a single intensity threshold could be applied to all images (absolute intensity 500).

All images were processed using the CellProfiler software (Carpenter et al., 2006). The processing pipeline is summarized here and is available from the authors upon request. Briefly, the pipeline first generated VGLUT3 objects from the VGLUT3 grayscale image using the pre-determined intensity threshold. Then the final thresholded, binary TPH2 image output from the FIJI macro was used to generate a single TPH2 object for each individual cell. The TPH2 and VGLUT3 images were re-matched to each other and the VGLUT3 objects were assigned to corresponding TPH2 objects based on overlapping area. Measurements were then performed to calculate the grayscale intensity of the full images and of the identified objects. The size and shape of TPH2 and VGLUT3 objects were determined. The area of VGLUT3 signal that lay within and outside of the TPH2 objects was also calculated. If greater than or equal to 20 pixels of a TPH2 cell were above the VGLUT3 threshold, that cell was classified as VGLUT3+. We found that there was a large proportion of VGLUT3+ cells with <50 pixels above the VGLUT3 threshold, and so we determined a second cutoff to identify highly VGLUT3+ TPH2+ cells where greater than or equal to 50 pixels were above the threshold.

### 2.9. Statistical analysis

Statistical analysis of TPH2+ cell measures were performed using R (Wickham et al., 2019; R Core Team, 2021). This study utilized mixed effect/multi-level modeling (MLM) to investigate effects of maternal and post-weaning diet and sex on outcome measures of interest. Measurements were calculated per cell and then per-image averages were generated for each image. The MLM modeling framework was selected as it allows examination of parameters that vary at different levels and allows data to be organized in nested levels. In this study the multiple images collected from each subregion were “nested” within every animal. Normality was assessed using skewness and kurtosis scores as well as the Shapiro–Wilkes test with a *p* > 0.05 considered normally distributed. The following measures were found to be normally distributed after the indicated transformation was applied: TPH2+ cell density (square-root), TPH2+ cell area (square-root), TPH2+ mean intensity (square-root), and VGLUT3+ cell density (square-root). Additionally, Bonferroni corrections were performed whenever multiple comparisons were conducted.

Model creation for the final analysis included fixed effects of diet and sex, and random effects were introduced to the models to account for the random variability in the number of images collected from each animal and nucleus within that animal. As not every animal contributed an image for each subnucleus identified, the random effects of the model are considered partially crossed and not “intrinsically nested.” To address the unequal representation of animals and subnuclei, we included a partially crossed random effect to control for the variation in the number of images derived from each. The model was run with the ImerTest package in R (Kuznetsova et al., 2017). The model formula that was selected for final analysis was:

TPH2 Cell Measure ∼ Diet Group + Sex + (1| Offspring ID) + (1| Offspring ID: Nuclei)

In this model, *TPH2 Cell Measure* is the outcome variable, *Diet Group* and *Sex* are the fixed effects, and the components *(1| Offspring ID)* and *(1| Offspring ID: Nuclei)* indicate the random effects. In the model, the syntax “(1| effects)” indicates that the effects are allowed to have randomly varying intercepts at each level of the effect, while the slope is held constant. The syntax (*Offspring ID: Nuclei*) indicates these two random variables are partially crossed (i.e., not every animal contributed an image from every nuclei). Thus, this model framework is based on the expectation that the relationship between diet group, sex, and cell density remains constant (fixed slopes) while being able to account for the fact that some nuclei may be predisposed to having inherently different levels of cell density than other nuclei (random intercept). Similarly, including (*1| Offspring ID*) as a random variable allows for the model to account for individual differences that are unrelated to diet group or sex effects. Model building began with an intercept model and sequentially added fixed and random effects based on *a priori* hypotheses of how each factor would influence the data. The best fitting model was determined using a variety of model fit metrics including Psuedo-R2 values, Intra-class correlation coefficients (ICC), Bayesian information criterion (BIC), and Akaike information criterion (AIC) with decreasing BIC and AIC values indicating better fit.

When creating models to examine differences between subnuclei, we included nuclei as a fixed effect with the same random effects as described previously for the intervention model. The final model used for this analysis was:

TPH2 Cell Measure ∼ Nuclei + (1| Offspring ID) + (1| Offspring ID: Nuclei)

Vesicular glutamate transporter 3 staining revealed punctate signal that may be indicative of the vesicles containing glutamate. Due to the binary nature in signal, we thresholded the VGLUT3 images and performed additional analyses on the area of VGLUT3 images above this threshold (section “2.8 Image processing”). These measurements were non-parametric due to data being heavily skewed because of a significant number of images containing no VGLTU3 staining and were thus investigated through a two-step analysis. First, we converted these data to a binary dataset where an image either contained VGLUT3 staining or did not. This transformed data was analyzed utilizing logistic regression analysis within the MLM framework as described previously. We then analyzed VGLUT3 data in a second method where only images that contained VGLUT3 staining were included in analysis as this data was normally distributed, utilizing similar MLM methodology. The combination of these two methods allowed for the comprehensive assessment of the variance structure of this data set that was heavily inflated and skewed by zeros.

## 3. Results

### 3.1. TPH2 and VGLUT3 characterizations

#### 3.1.1. TPH2 characterization

Tryptophan hydroxylase 2+ cell distribution and characteristics have been examined extensively in rodents, to our knowledge this study is the first to describe the characteristics of TPH2+ cells within subnuclei of the DR in a non-human primate model. While highly correlated, the DR subnuclei appear differently in rodents and direct equivalences have not been made (Hioki et al., 2010; Calizo et al., 2011; Haugas et al., 2016; Ren et al., 2019). Our detailed characterization will provide a valuable resource to research groups investigating the development of the DR in primates.

We compared TPH2+ cell density, size, and integrated intensity for eight different subnuclei across the rostro-caudal extent of the serotonergic raphe nuclei. The eight subnuclei were chosen from the 9 total bounding regions collected that had a high enough sampling rate to produce meaningful results (pontine raphe excluded). In addition to the qualitative distinctions described in the methods (section “2.7 Subnuclei division”), the subnuclei stratify on distinguishing features such as cell size and TPH2 cell density (data not shown).

**FIGURE 2:**
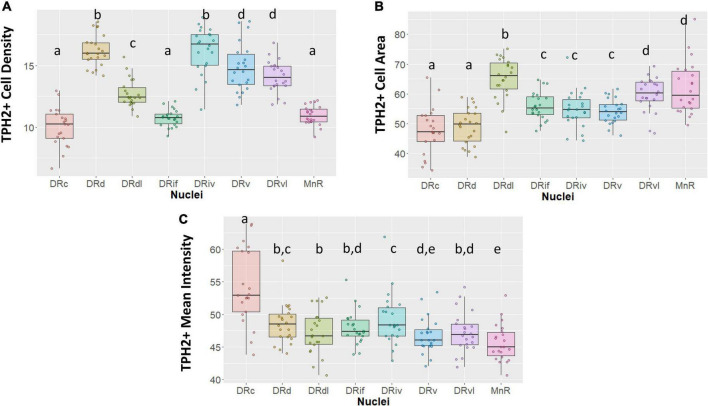
Tryptophan hydroxylase 2 (TPH2) cell measurements across subnuclei within the greater raphe nuclei. Data are expressed as box plots with boxes indicating the 1st–3rd quartile range and the median expressed as the horizontal bold line. Nuclei that share letters (e.g., “a”) are not significantly different from each other, different letters indicate significant differences between subnuclei at *p* < 0.05. Bonferroni corrections were made when making multiple comparisons across all different subnuclei included. In instances denoted with multiple letters (e.g., “a, b”) these groups are not significantly different from any groups that are also denoted with the same letter. **(A)** Number of TPH2+ cells in each subnuclei region. **(B)** Cell area in pixels of TPH2+ cells. **(C)** Mean intensity of TPH2 signal per image. DRd, dorsal nucleus of the dorsal raphe; DRdl, dorsolateral nucleus of the dorsal raphe; DRv, ventral nucleus of the dorsal raphe; DRvl, ventrolateral nucleus of the dorsal raphe; DRiv, fourth nerve nucleus of the dorsal raphe; DRif, interfascicular nucleus of the dorsal raphe subnucleus; MnR, median raphe nucleus.

Our morphological and TPH2 characterization support the distinction of eight subnuclei (Figure 2). For example, although DRd appears to transition into the DRc as sections progress caudally, the two nuclei achieve TPH2 abundance in different ways. Whereas the DRd exhibits the most densely packed TPH2 cells (*p* < 0.05, Figure 2A), what the DRc lacks in cell density, it makes up for in TPH2 signal intensity (*p* < 0.05, Figure 2C).

Further, our designation of the rostral DRdl as a separate subnucleus from the DRd was supported by a stronger homogeneity between the two lateral wing nuclei than between the DRdl and DRd. The DRdl and the DRvl exhibited elevated mean TPH2 intensity and cell size with respect to their medial counterparts (*p* < 0.05, Figures 2B, C). Nevertheless, the two lateral wings were more than just separated spatially; the DRdl TPH2 cells were significantly larger and less densely packed than DRvl TPH2 cells (*p* < 0.05, Figures 2A, B).

The DRif, which is uniquely identified in primates but arguably is homologous to the larger DRv in rodents, did in fact possess similar cell size and TPH2 intensity as the DRv (*p* > 0.05, Figures 2B, C). Nonetheless, the uniformly oriented fusiform cells of the DRif distinguish it from the more diverse and compact DRv (*p* < 0.05, Figure 2A). The DRiv displayed similar characteristics to its most proximal subnuclei (DRv) but did show minor yet significantly increased cell density and mean TPH2 signal (*p* < 0.05, Figures 2A, C).

Our data indicate that there is an unavoidable difference between the rostral and medial raphe and the caudal subnuclei–the DRc and MnR (*p* < 0.05, Figures 2A, C). Finally, the MnR displayed the lowest mean intensity of the subregions yet it contained some of the largest sized cells, consistent with its distinct developmental origin (*p* < 0.05, Figure 2C).

#### 3.1.2. VGLUT3 characterization

**FIGURE 3:**
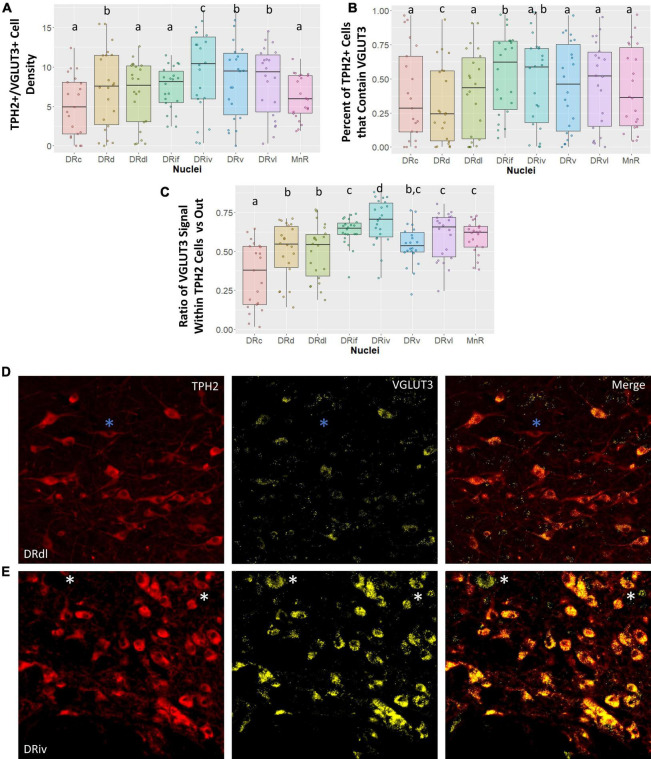
Vesicular glutamate transporter 3 (VGLUT3) cell measurements across subnuclei within the greater raphe nuclei. Data are expressed as box plots with boxes indicating the 1st–3rd quartile range and the median expressed as the horizontal bold line. Nuclei that share letters (e.g., “a”) are not significantly different from each other, different letters indicate significant differences between subnuclei at *p* < 0.05. Bonferroni corrections were made when making multiple comparisons across all different subnuclei included. In instances denoted with multiple letters (e.g., “a, b”) these groups are not significantly different from any groups that are also denoted with the same letter. **(A)** Cell density of tryptophan hydroxylase 2 (TPH2)/VGLUT3 dual positive cells in each subnuclei region. Dual positive cells were defined as TPH2+ cells that contained 20 or more pixels of VGLUT3 signal. **(B)** Percent of all TPH2+ cells that also have VGLUT3 signal. **(C)** Ratio of signal that is located either inside or outside of a TPH2+ cell. A ratio of 0.5 indicates VGLUT3 signal is equally likely as not to occur within a TPH2+ cell. Ratios above 0.5 indicate a high likelihood VGLUT3 signal will be located inside a TPH2+ cell. **(D,E)** Representative images of TPH2 and VGLUT3 signal localization. **(E)** Typical VGLUT3 signal in the DRdl. Blue asterisks indicate TPH2+ cells with a lack of VGLUT3 signal present. Example of intense VGLUT3 signal found in the DRiv. White asterisks indicate VGLUT3+ non-TPH2 cells. These cells additionally serve to demonstrate that although TPH2 signal and VGLUT3 signal are correlated, this is not the result of “bleed-through” between channels. DRd, dorsal nucleus of the dorsal raphe; DRdl, dorsolateral nucleus of the dorsal raphe; DRv, ventral nucleus of the dorsal raphe; DRvl, ventrolateral nucleus of the dorsal raphe; DRiv, fourth nerve nucleus of the dorsal raphe; DRif, interfascicular nucleus of the dorsal raphe subnucleus; MnR, median raphe nucleus.

To our knowledge, VGLUT3 abundance in the subnuclei of the DR has previously only been studied in rodent models and this is the first study to characterize VGLUT3 staining in subnuclei of the primate raphe. Here we examined four different measures of VGLUT3 staining in the eight raphe subnuclei: (1) the number of cells that were dual stained for both VGLUT3 and TPH2 (VGLUT3+/TPH2+); (2) the proportion of all TPH2+ cells that contained VGLUT3 staining; and (3) the proportion of VGLUT3 signal located within the TPH2+ cells of an image versus outside of those cells but within the image boundary. An example of a TPH2+ cell that does not contain VLGUT3 signal is denoted by blue asterisks in Figure 3D. An example of VGLUT3 signal occurring outside of a TPH2+ cell can be seen in Figure 3E denoted by white asterisks. We found that classifying cells as highly VGLUT3+ did not reveal any additional information not present in our standard VGLUT3 density measurement.

Vesicular glutamate transporter 3 signal is highly variable across different subnuclei in the raphe nuclei (Figure 3). Our data demonstrate that a large amount of variability in VGLUT3 measurements is yet unaccounted for. In spite of this, we uncovered striking differences between the DRiv and all other subnuclei. This region has the highest density of VGLUT3+/TPH2+ cells, the largest proportion of TPH2 cells that are VGLUT3+, and the most VGLUT3 signal localized within TPH2+ cells (Figures 3A–C). Examples of this enrichment are clearly seen in Figures 3D, E.

The VGLUT3 measurements reinforced the divide between the caudal subnuclei and the rest. The MnR has the lowest VGLUT3+ density and the fewest number of TPH2+ cells that are VGLUT3+, but nevertheless the VGLUT3 signal that is present is quite specific to the TPH2 neurons (Figure 3C). This contrasts with the VGLUT3 localization in the DRc, which was significantly less than the other nuclei and below chance. The opposite is true in the DRd, which is often considered a more medial extension of the DRc. Despite the high TPH2 cell density in the DRd, the chances of finding a VGLUT3+/TPH2+ cell in the DRd was actually lower than in the DRc, a comparatively sparse region (Figure 3B). Yet, the little VGLUT3 signal present in DRd images was more likely to be found within TPH2 cells than outside of them, still indicating a relative VGLUT3 enrichment in these cells (Figure 3A).

Lastly, we found that the DRvl possessed more VGLUT3+/TPH2+ cells per unit area than the DRdl (Figure 3A). However, the lack of difference in other VGLUT3 measures suggests that this may be an artifact of the overall increased TPH2 density in the DRvl.

### 3.2. Effect of Western-style diet on TPH2 and VGLUT3 measures

For analyses examining the effects of WSD on TPH2 and VGLUT3 cell measurements, we focused our analysis on five subnuclei that the rodent literature most strongly associates with projection sites in the forebrain (Ren et al., 2018). These subnuclei include the DRd, DRdl, DRv, DRvl, and DRif. We also examined the MnR as our previous results found that post-weaning diet altered *tph2* mRNA abundance in that region (Thompson et al., 2017).

#### 3.2.1. TPH2 measures

##### 3.2.1.1. TPH2+ cell count

**FIGURE 4:**
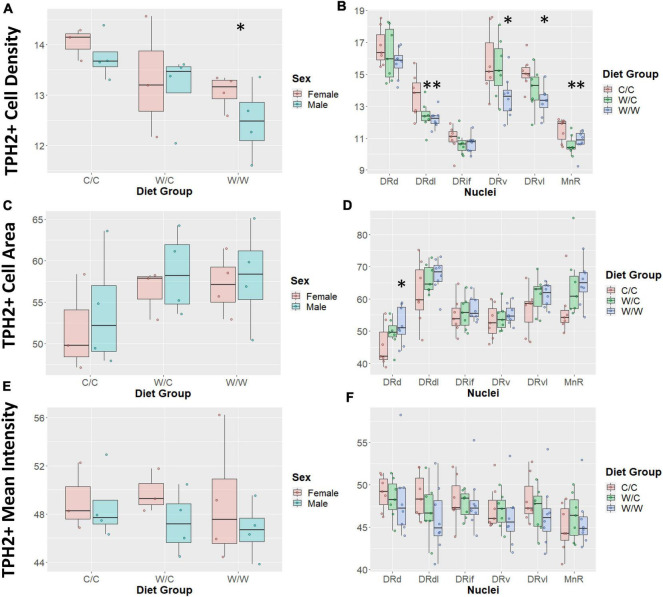
Western-style diet (WSD) influences raphe tryptophan hydroxylase 2+ (TPH2+) cell outcomes. Data are expressed as box plots with boxes indicating the 1st–3rd quartile range and the median expressed as the horizontal bold line. Asterisks indicate significant differences compared to C/C groups. **(A)** TPH2 cell count was decreased by perinatal exposure to WSD. The W/W group was decreased compared to the C/C and W/C groups. **(B)** TPH2 cell number in specific regions appear to be uniquely impacted by perinatal WSD. The DRd and DRif appears not to be impacted, while the DRdl and MnR subregions both the W/C (*p* < 0.05) and W/W (*p* < 0.05) groups were reduced compared to the C/C group. Additionally, in the DRv and DRvl subregions only the W/W was reduced compared to the C/C group (*p* < 0.05). While the W/C group was not significantly different from the C/C group, it did trend in the same direction as the W/W group. **(C)** TPH2 cell area was not found to be significantly different between diet groups. **(D)** TPH2 cell area was only significantly increased in the DRd subregion (*p* < 0.05). **(E)** TPH2 mean intensity per image was not found to be significantly different between diet groups. **(F)** TPH2 mean intensity per image was not found to be significantly different in any specific subregions. DRd, dorsal nucleus of the dorsal raphe; DRdl, dorsolateral nucleus of the dorsal raphe; DRv, ventral nucleus of the dorsal raphe; DRvl, ventrolateral nucleus of the dorsal raphe; MnR, median raphe nucleus.

Diet had an overall effect on TPH2+ cell density such that the TPH2+ population was least concentrated in the W/W group (β = −1.09, *p* < 0.05; Figure 4A), with the W/C group following a similar trend (β = −0.72, *p* = 0.19). We did not observe an overall sex effect (β = −0.31, *p* = 0.43) on TPH2+ cell density.

Since density was highly variable between DR compartments, we examined the impact of WSD on the specific subnuclei. In this analysis we found that the pan-DR effect of W/W on TPH2 cell density was reflected in the DRv, DRdl, and DRvl, all of which were significantly decreased from C/C (β = −1.35, *p* < 0.05). The diet intervention (consumption of a CTR diet at weaning) did not correct TPH2 density impairments in the DRdl (β = −1.23, *p* < 0.05). Intriguingly, the MnR was also sensitive to the effect of WSD on TPH2 cell density, with the TPH2 cell density of W/C (β = −0.89, *p* < 0.05) and W/W (β = −0.74, *p* < 0.05) groups decreased compared to the C/C group (Figure 4B). Additionally, a sex effect was observed in the MnR, with male animals having reduced TPH2+ cell density compared to females (β = −0.62, *p* < 0.05).

##### 3.2.1.2. TPH2+ cell size

We found no overall effect of diet group on TPH2+ cell size in the raphe as a whole (*p* > 0.1; Figure 4C). Additionally, we did not observe an overall sex effect (β = 1.71, *p* = 0.44) on TPH2+ cell size (Figure 4C). As we saw significant differences in cell density at the nuclei level, we asked whether diet elicited a subnuclei-specific effect on TPH2+ cell size. We found a significant increase in TPH2+ cell size in the DRd nuclei of the W/W (β = 6.63, *p* < 0.05), but not the W/C group (β = 3.76, *p* = 0.25) compared to controls (Figure 4D). We did not find significant differences in cell size for any other subnuclei.

##### 3.2.1.3. TPH2 mean intensity

We found that diet did not have an overall effect on TPH2 mean intensity in the raphe as a whole (*p* > 0.5; Figure 4E). Similar to other measures, we observed large variability in mean intensity levels across subnuclei and probed whether diet impacted specific subnuclei. In this case, we found that diet did not account for variability in TPH2 intensity for any subnuclei (Figure 4F).

#### 3.2.2. VGLUT3 measures

**FIGURE 5:**
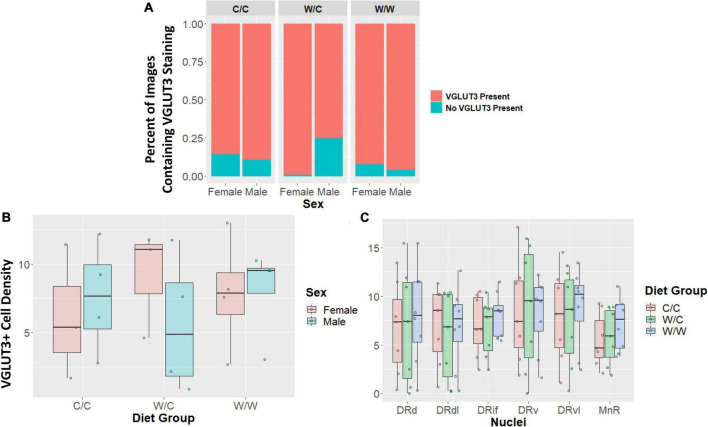
Vesicular glutamate transporter 3 (VGLUT3) cell density does not appear to be impacted by maternal Western-style diet (WSD). Data in panel **(A)** is expressed as percentages of images containing either some or no VGLUT3 staining. Data in panels (B/C) are expressed as box plots with boxes indicating the 1st–3rd quartile range and the median expressed as the horizontal bold line. **(A)** No significant diet or sex main effects were found when comparing the percentage of images that either contained some VGLUT3 staining or no staining at all. **(B)** VGLUT3 cell density was not significantly impacted by perinatal diet. **(C)** VGLUT3 cell density in specific subregions were not uniquely impacted by perinatal diet. DRd, dorsal nucleus of the dorsal raphe; DRdl, dorsolateral nucleus of the dorsal raphe; DRv, ventral nucleus of the dorsal raphe; DRvl, ventrolateral nucleus of the dorsal raphe; MnR, median raphe nucleus.

The VGLUT3+ identity of TPH2+ cells were not impacted by perinatal diet for any measure examined. The probability of having an image with zero staining or some VGLUT3 was not significantly different for the W/C (β = 0.07, *p* = 0.48), or W/W (β = −0.06, *p* = 0.53) groups when compared to controls (Figure 5A). Additionally, when we analyzed only images that contained at least some VGLUT3+ staining we found no significant difference in cell number for W/C (β = −0.11, *p* = 0.95), or W/W (β = 0.64, *p* = 0.73) animals compared to control animals (Figure 5B). Further, we found that no specific nuclei were impacted by diet (Figure 5C). This pattern of results was found to be similarly non-significant for both the proportion of VGLUT3+ TPH2 cells and the quantity of highly VGLUT3 expressing cells (Supplementary Figures 1–3).

## 4. Discussion

We investigated how TPH2 cell measurements varied throughout the subnuclei of the DR of juvenile Japanese macaques. TPH2 is the rate-limiting enzyme for serotonin production, and the TPH2 neurons in the soma of DR neurons generate the vast majority of the serotonin for the entire central nervous system. It is known that serotonergic anatomy is established in the early postnatal period in rodents (Alonso et al., 2013; Deneris and Gaspar, 2018). This is the first study to systematically examine the DR anatomy in juvenile primates. We found that the anatomical distribution of TPH2 neurons in the DR of juvenile macaques was similar to that of adult Japanese macaques (Bethea and Reddy, 2012), rhesus and cynomolgus macaques (Wilson and Molliver, 1991; Sanchez et al., 2005), and humans (Baker et al., 1990). Our analysis suggests that the anatomical and morphological properties of the adult DR are established before 13 months of age in Japanese macaques.

One of the major goals of this study was to reconcile the anatomical differences in TPH2 neuron clusters in the DR between rodents and primates. Much of the research into the functional properties of the DR comes from rodent studies, particularly the determination of the targets of DR projections [reviewed in Hale and Lowry (2011); Waselus et al. (2011); Commons (2020)]. Translatability of these findings is obscured by some key differences in the anatomy of the rodent and primate DR. Rodents have a considerable serotonergic neuron population along the midline of the dorsal and DRv, while in primates the DRd has clear clusters on the left and right hemispheres. In mice and rats, most dorsal serotonergic neurons are in the midline population and these cells are significantly smaller than the ventral population (Calizo et al., 2011). In contrast, our analysis and previous studies have shown that the largest neurons in the primate DR are in the DRdl (Baker et al., 1990; Wilson and Molliver, 1991). Importantly, it is unclear which primate DR subregion is homologous to the DRv in rodents.

To better direct translational research in the developing serotonergic system, we examined if VGLUT3 expression provided insight with which we can better compare the murine and primate DR subnuclei. This study is the first to detail VGLUT3 expression patterns in the primate DR. We found considerable VGLUT3 signal within (somatic) and outside of TPH2 neurons throughout the DR, consistent with the rodent literature (Gras et al., 2002; Hioki et al., 2010; Ren et al., 2018).

The degree of overlap reported in TPH2 and VGLUT3 expressing neurons varies based on methodology and host species. We found that in the juvenile macaque DR, roughly 45% of TPH2 neurons were VGLUT3 positive. One study in adult rats estimated that about 30% of serotonergic neurons in the DR were VGLUT3+ (Haugas et al., 2016). In spite of a lack of agreement on the proportion of VGLUT3+ serotonergic neurons in the DR, what remains clear is that different DR populations possess unique proportions of VGLUT3 and TPH2 colocalization (Hioki et al., 2010; Okaty et al., 2015; Haugas et al., 2016; Huang et al., 2019). Recently, one research group found that cortical regions are primarily targeted by VGLUT3+ serotonergic neurons, regardless of DR subnuclei (Ren et al., 2019). There is an additional degree of subnuclei specificity; as they reported that in the DRv roughly 60% of VGLUT3 neurons overlapped with TPH2 neurons, and that these populations have specific cortical targets (Ren et al., 2018).

We hypothesized that perinatal WSD would decrease TPH2 availability across the raphe. TPH2 availability is limited by the concentration of TPH2 in each cell (estimated by mean TPH2 signal intensity), the amount of TPH2 within each cell (estimated by TPH2+ cell size), and the number of TPH2-producing cells (evaluated with TPH2+ cell density). We found that the DR of animals that received nutrition from WSD *in utero* through to sample collection presented with fewer TPH2 neurons. These results were driven by significant decreases in TPH2 cell density in the DRdl, DRvl, DRv, and MnR. Surprisingly, we did find that W/W increased TPH2 cell size in the DRd. Our findings indicate that perinatal WSD likely limits the serotonin production capacity of the DR.

Based on our previous findings that W/C and W/W decreased the percent area of *Tph2* mRNA in the DR (Thompson et al., 2017), and the corroboration of those findings in the W/W in this study, we expected that diet intervention would not reverse the insult to TPH2 neuron size and density. However, we found that TPH2 cell density was less susceptible to WSD exposure when limited to the pre-weaning period, and that only the DRdl and the MnR exhibited a significant decrease in TPH2 cell density. Intriguingly, TPH2 cell density in the MnR was the only measure found to be different between male and female animals. More work is needed to comprehensively understand sex differences expressed in the raphe nuclei.

Overall, our data indicate that TPH2 availability is sensitive to perinatal WSD, with the DRdl and MnR exhibiting the most long-lasting effects even after post-natal diet intervention (switching onto the CTR diet). Given that the TPH2 neurons in the DRdl are the largest, this reduction could have important implications for the downstream brain regions and behaviors. Combined with our previous findings (Thompson et al., 2017), these data indicate that WSD-induced changes in the density of TPH2 neurons can restrict serotonin delivery to target brain regions and contribute to dysregulated physiological and behavioral stress response. This conclusion is supported by evidence from murine models that indicate TPH2 abundance is associated with anxiety-like behaviors and is particularly sensitive to early life perturbations (Gardner et al., 2009; Donner et al., 2012; Donner et al., 2018).

Evidence from a rodent model demonstrated that a maternal inflammatory insult produced the same phenotype of reduced TPH2 cell number in adult offspring as well as decreased serotonergic availability in target brain regions (Hsueh et al., 2017) supporting our hypothesis of WSD-induced inflammation being an underlying mechanism for the changes in the serotonergic system. These results are consistent with our evidence from our non-human primate model where we have previously shown that elevated maternal inflammatory response mediates the influence of WSD on offspring behavioral response (Thompson et al., 2018). Our present results suggest that these changes could be alleviated if nutritional balance is restored before adulthood.

We predicted that the compensatory changes in VGLUT3 expression may coincide with TPH2 disturbances, but no dietary effect reached significance in any measure examined. These results are consistent with a study in VGLUT3 knockout mice where VGLUT3 deletion did not modify TPH2 level or serotonin levels (Amilhon et al., 2010). This study did, however, find that somatic VGLUT3 regulated basal neuronal activity of serotonergic neurons by facilitating glutamate-dependent serotonin reuptake. Whether the differences in VGLUT3 expression between DR regions contribute to nuclei-specific basal activity rates has not been examined.

Our study was limited by a small sample size and a large number of factors (i.e., maternal diet, post-weaning diet, sex, and possibly differences in maternal metabolic state) which, in combination with mostly non-parametric distribution of measurements, limited our statistical analyses and power. Additionally, another limitation was not having a group where animals experienced a maternal CTR diet and were switched to a WSD post-weaning. Addition of this group could help determine whether a post-weaning WSD independently influenced certain subnuclei, or whether it was the combination of a pre- and post-weaning WSD that was associated with differences in TPH2 neuron characteristics. Future investigation will pursue consideration of maternal metabolic state covariates (e.g., maternal age and pre-pregnancy obesity) that have been shown in previous work to significantly impact offspring serotonergic and behavioral outcomes. Follow-up experiments will attempt to both apply and validate conclusions drawn in this analysis to inform future DR subnuclei groupings.

In summary, the juvenile Japanese macaque DR reflects similar complexity and heterogeneity to the adult macaque and human DR. Our examination reveals that VGLUT3 is broadly co-expressed in TPH2 neurons in the DR of non-human primates. Differential VGLUT3 expression in juvenile macaques mirrors patterns seen in rodents, where VGLUT3 participates in serotonergic transmission to cortical and limbic areas and modulates behavioral response. Importantly, maternal WSD and continued WSD consumption post-weaning decreased TPH2 availability and resulted in nuclei-specific alterations in TPH2 protein expression in the primate DR. Additionally, the observed subnuclei-specific effects may impact serotonergic innervation of certain projection locations in the forebrain and could provide insight into how WSD exposure may program offspring risk for anxiety and stress responses.

## Data availability statement

The raw data supporting the conclusions of this article will be made available by the authors, without undue reservation.

## Ethics statement

The animal study was reviewed and approved by the Oregon National Primate Research Center’s Institutional Animal Care and Use Committee.

## Author contributions

ES conceived the project and designed the research. JT and AM performed the experiments. JT designed FIJI macros. GD, AM, and ES analyzed the data. GD, JT, and ES wrote the manuscript with contributions from all authors. All authors discussed the data.
