# Supplementary material for: Perinatal Western-style diet alters serotonergic neurons in the macaque raphe nuclei
Source: Front Neurosci. 2023 Jan 10;16:1067479. doi: 10.3389/fnins.2022.1067479 (PMC9872117; doi:10.3389/fnins.2022.1067479)
Supplement: Supplementary file 1 [file Data_Sheet_1.docx]

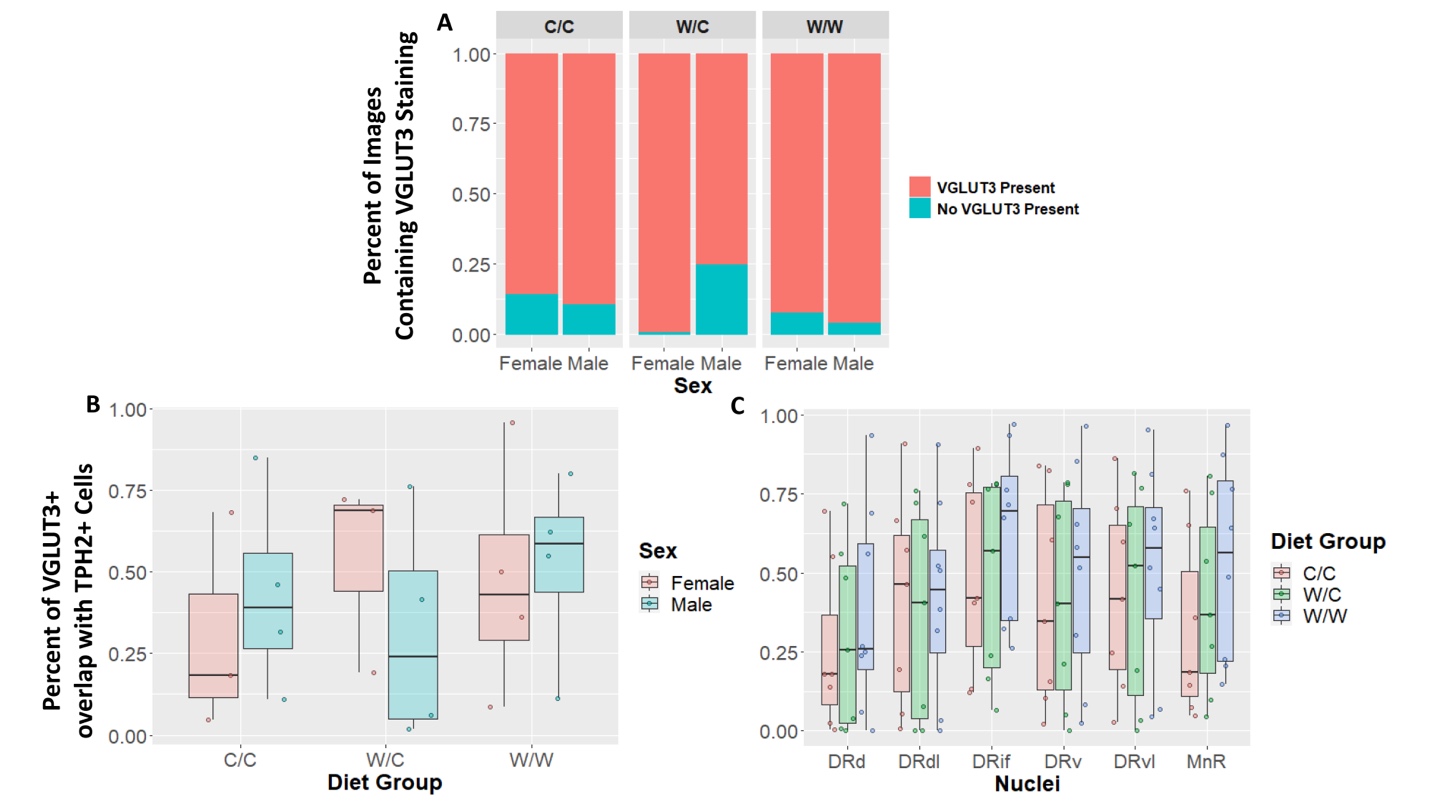


**Supplementary Figure 1.** Proportion of VGLUT3+/TPH2+ cells does not appear to be impacted by maternal WSD. Data in B and C are expressed as box plots with boxes indicating the 1^st^ to 3^rd^ quartile range and the median expressed as the horizontal bold line.

A) No significant diet or sex main effects were found when comparing the percentage of images that either contained some VGLUT3 staining or no staining at all.

B) Proportion of VGLUT3+/TPH2+ cells was not significantly impacted by perinatal diet.

C) Proportion of VGLUT3+/TPH2+ cells in specific subregions were not uniquely impacted by perinatal diet.

Abbreviations: DRd: dorsal nucleus of the Dorsal Raphe; DRdl: dorsolateral nucleus of the Dorsal Raphe DRv: ventral nucleus of the Dorsal Raphe; DRvl: ventrolateral nucleus of the Dorsal Raphe MnR: Median Raphe nucleus

**
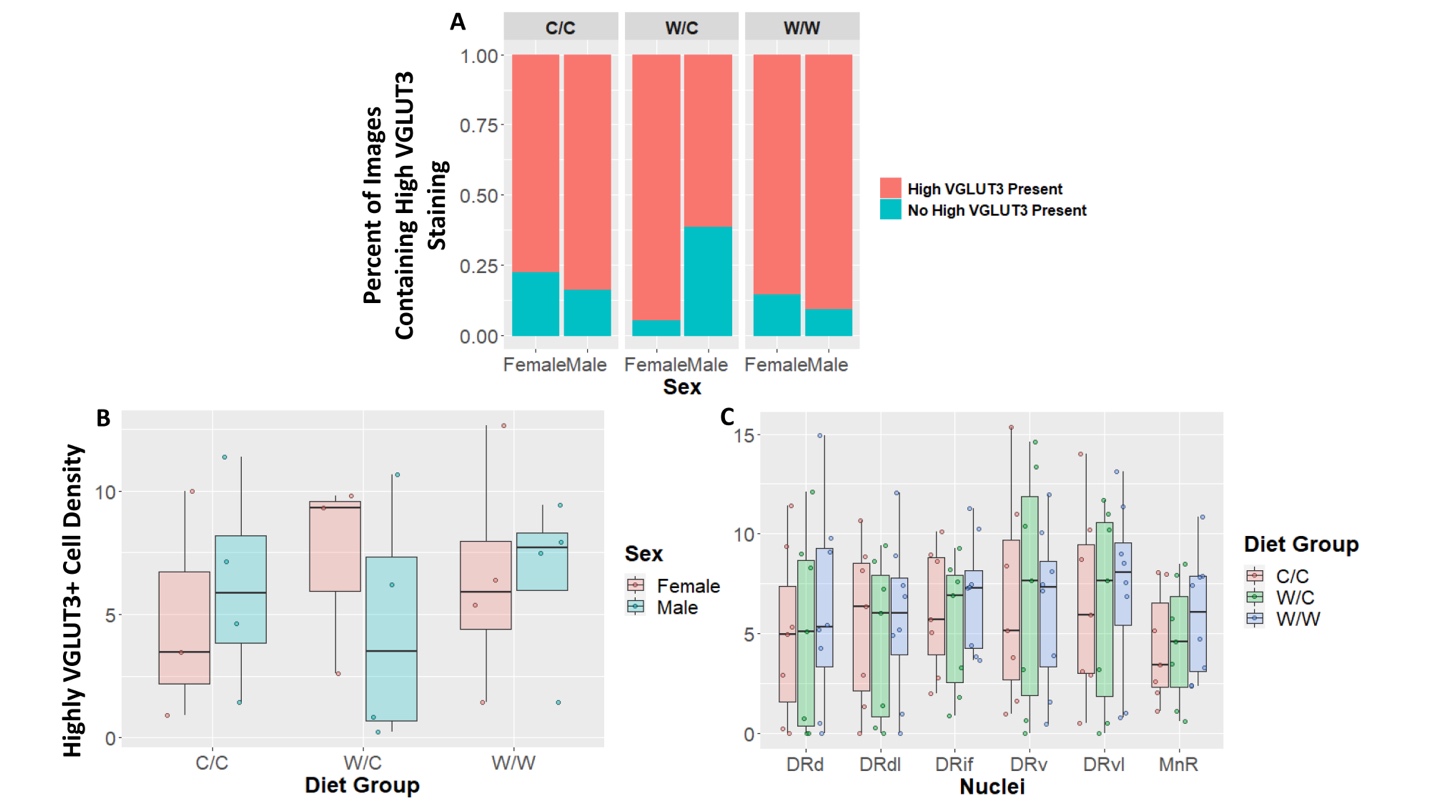
Supplementary Figure 2.** Cell density of highly VGLUT3+ cells does not appear to be impacted by maternal WSD. Data in B and C are expressed as box plots with boxes indicating the 1^st^ to 3^rd^ quartile range and the median expressed as the horizontal bold line.

A) No significant diet or sex main effects were found when comparing the percentage of images that either contained some highly VGLUT3+ stained cells or no highly VGLUT3+ stained cells at all.

B) Cell density of highly VGLUT3+ cells was not significantly impacted by perinatal diet.

C) Cell density of highly VGLUT3+ cells in specific subregions were not uniquely impacted by perinatal diet.

Abbreviations: DRd: dorsal nucleus of the Dorsal Raphe; DRdl: dorsolateral nucleus of the Dorsal Raphe DRv: ventral nucleus of the Dorsal Raphe; DRvl: ventrolateral nucleus of the Dorsal Raphe MnR: Median Raphe nucleus

**
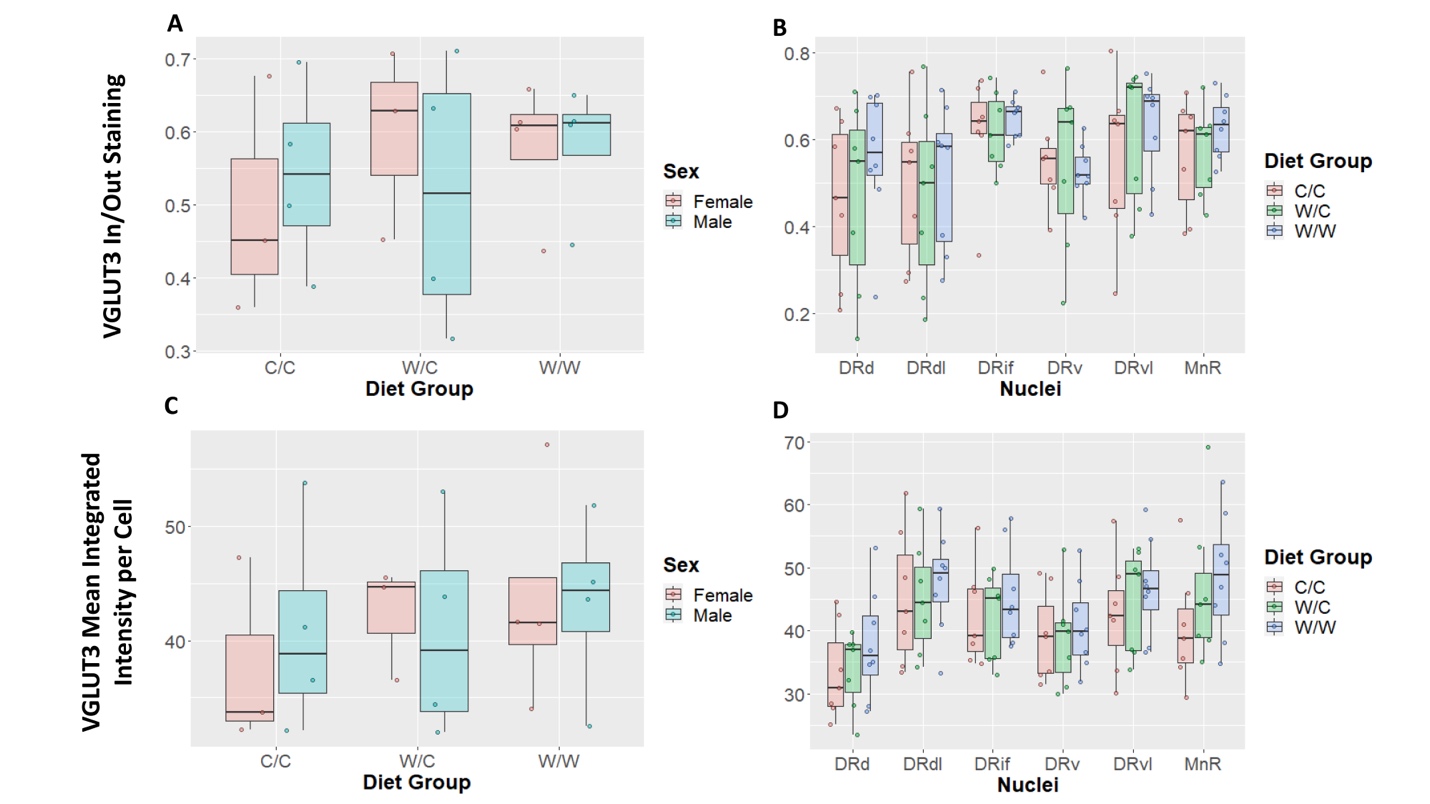
Supplementary Figure 3.** WSD does not appear to influence other raphe VGLUT3+ cell outcomes. Data are expressed as box plots with boxes indicating the 1^st^ to 3^rd^ quartile range and the median expressed as the horizontal bold line.

A) Ratio of signal that is located either inside or outside of a TPH2+ cell. A ratio of 0.5 indicates VGLUT3 signal is equally likely as not to occur within a TPH2+ cell. Ratios above 0.5 indicate a highly likelihood VGLUT3 signal will be located inside a TPH2+ cell. VGLUT3 In/Out ratio staining was not found to be significantly impacted by perinatal exposure to WSD.

B) VGLUT3 In/Out ratio staining was not found to be significantly different in any specific subregions.

C) VGLUT3 average cell integrated intensity was not found to be significantly different between diet groups.

D) VGLUT3 average cell integrated intensity was not found to be significantly different in any specific subregions.

Abbreviations: DRd: dorsal nucleus of the Dorsal Raphe; DRdl: dorsolateral nucleus of the Dorsal Raphe DRv: ventral nucleus of the Dorsal Raphe; DRvl: ventrolateral nucleus of the Dorsal Raphe; MnR: Median Raphe nucleus
